# Pan genome clustering identifies a novel mosaic prophage specific to *Salmonella* Enteritidis lineage associated with the invasive disease in India

**DOI:** 10.1371/journal.pntd.0013113

**Published:** 2026-08-31

**Authors:** Jobin John Jacob, Aravind Velmurugan, Dhanalakshmi Solaimalai, Ramya Iyadurai, Karthik Gunasekaran, Jansi Rani Malaiyappan, M. Yesudoss, M. Vasuki, Binesh Lal, Jacob John, Kamini Walia, Balaji Veeraraghavan

**Affiliations:** 1 Department of Clinical Microbiology, Christian Medical College, Vellore, India; 2 Department of Medicine Unit V, Christian Medical College, Vellore, India; 3 Department of Community Medicine, Christian Medical College, Vellore, India; 4 Division of Descriptive Studies, Indian Council of Medical Research, New Delhi, India; University of Connecticut College of Agriculture Health and Natural Resources, UNITED STATES OF AMERICA

## Abstract

*Salmonella enterica* serovar Enteritidis is a leading cause of invasive non-typhoidal Salmonella (iNTS) disease globally, particularly in sub-Saharan Africa. In contrast, the epidemiology and population structure of invasive *S*. Enteritidis in South Asia remain poorly characterized. This study investigates the clinical presentation, phylogenetic relationships and genomic characteristics of *S*. Enteritidis bloodstream infections (BSIs) in India. Clinical data were collected from 101 patients with *S*. Enteritidis BSI between 2012 and 2022. Whole-genome sequencing was performed on representative bloodstream isolates together with isolates from non-blood clinical specimens and poultry sources. Comparative genomic analyses included phylogenetic reconstruction, invasiveness index prediction, and prophage characterization. Infants and immunosuppressed individuals were disproportionately affected by iNTS disease. Phylogenetic analysis identified four major lineages of *S*. Enteritidis. Most BSI isolates clustered in a previously unrecognized lineage, designated the Global Intermediate Clade, which occupied a phylogenetic position between the Global outlier and Global epidemic clades. Bayesian inference dated its most recent common ancestor to around 1789 AD (95% HPD: 1692–1941), with global circulation confirmed by European and Asian isolates. The Global Intermediate clade exhibited the second-highest invasiveness index (median 0.221, SD 0.013) after the West African clade; however, this index reflects genomic signatures associated with invasiveness and should not be interpreted as a direct measure of virulence. Poultry isolates clustered separately from the dominant bloodstream-associated lineage. Pan-genome analysis identified a lineage-specific mosaic prophage composed of modules homologous to prophages found in diverse Enterobacterales. This study provides the first detailed genomic insight into invasive *S*. Enteritidis in India and identifies a previously unrecognized Global Intermediate Clade associated with bloodstream infection. The distinct phylogenetic placement and genomic features of this lineage, including a lineage-specific mosaic prophage, warrant further investigation and support the need for expanded One Health genomic surveillance.

## Author summary

Invasive infections caused by *Salmonella enterica* serovar Enteritidis are a major public health concern in many low- and middle-income countries, yet their epidemiology in South Asia remains poorly understood. We analyzed clinical and genomic data from patients with bloodstream infections caused by *S*. Enteritidis in India and compared these isolates with strains obtained from poultry and publicly available global genomes. We found that invasive infections occurred most frequently in infants and individuals with immunosuppressive conditions. Genomic analysis identified a previously unrecognized lineage, which we named the Global Intermediate Clade, that accounted for a large proportion of bloodstream isolates in our collection. This lineage occupies a distinct position within the global *S*. Enteritidis population structure and carries a unique mosaic prophage not observed in other major lineages. Although the Global Intermediate Clade showed a comparatively elevated invasiveness index, this metric is based on genomic prediction and does not directly measure clinical virulence. Poultry isolates from our study were genetically distinct from the dominant bloodstream-associated lineage, although broader animal reservoirs cannot be excluded. Our findings expand current understanding of invasive *S*. Enteritidis in South Asia and highlight the value of integrated clinical and genomic surveillance for identifying and monitoring previously unrecognized pathogen lineages.

## Introduction

Nontyphoidal *Salmonella* (NTS) are a significant cause of gastrointestinal infections which are generally self-limiting and resolve without any clinical interventions [1,2]. However, over the past few decades, some serovars of NTS have emerged as a leading cause of BSI particularly in sub-Saharan African (sSA) countries [3,4]. As of 2017, sub-Saharan Africa accounted for 79% of the 535,000 global cases of invasive NTS (iNTS) disease and 85% of the 77,500 associated deaths [5]. iNTS infections are strongly associated with immunocompromised individuals, including those with HIV, malaria, malnutrition, and hematologic malignancies [6,7]. Clinical presentation often includes non-specific febrile illness, closely resembling typhoid fever or malaria, making diagnosis particularly challenging in resource-limited settings [8].

Although a wide range of *Salmonella* serovars have been reported to cause iNTS diseases, the majority of infections in sub-Saharan Africa are caused by two dominant serovars: *Salmonella enterica* serovar Typhimurium (ST313) and *Salmonella enterica* serovar Enteritidis (ST11) [9,10]. These endemic African lineages have been identified as the primary drivers of iNTS infections in the region [11]. Phylogenetic analysis based on single nucleotide polymorphisms (SNPs) has identified three distinct lineages (L1, L2, and L3) of *S*. Typhimurium ST313, which have evolved over time to exhibit increased human adaptation and invasiveness [12]. Similarly, the African clade of ST11 *S*. Enteritidis has been predicted to be more invasive than the Global epidemic clone and Global outlier cluster isolates [13].

While iNTS disease in sub-Saharan Africa has been studied extensively, isolates from other low-income settings have received less attention. In South Asian countries, the prevalence of iNTS has been largely underestimated due to limited surveillance data and underreporting [14,15]. Furthermore, the transmission dynamics of NTS serovars between humans, animals, and the environment in a One Health context remain poorly characterized [16]. Previously, a retrospective analysis of blood cultures collected at our tertiary care hospital in southern India region between 2000 and 2020 revealed that *S*. Typhimurium (49%) and *S*. Enteritidis (29%) were the most prevalent serovars causing iNTS infections [17]. Of these two, *S*. Typhimurium has been relatively well characterized, largely due to its use as an experimental model for human typhoid fever. However, the clinical presentation, antimicrobial resistance patterns, and genomic features of *S*. Enteritidis infections in our setting remain poorly documented.

In this study, we conducted a retrospective analysis of *S*. Enteritidis isolates obtained from BSI at a tertiary-care hospital in Vellore, India. Clinical characteristics and disease outcomes of these infections were also assessed. Additionally, *S*. Enteritidis isolates from livestock sources were included to investigate potential zoonotic transmission dynamics between humans and animals. Whole-genome sequencing (WGS) and comparative genomic analysis were performed to determine the phylogenetic relationships, genetic diversity, and invasive potential of these isolates within a global isolate collection. Furthermore, pan-genome analysis was performed to understand evolutionary trajectory, host adaptation mechanisms, and pathogenicity determinants of *S*. Enteritidis.

## Methods

### Ethics statement

Institutional Review Board (IRB) of Christian Medical College (CMC), Vellore, India gave ethical approval for this work vide IRB Min. No. 11878 dated 27^th^ February, 2019. As the required data had been collected as part of the standard of care for diagnosis, informed consent waiver was granted by the IRB committee Christian Medical College, Vellore

### Study design and data collection

This retrospective study was conducted between January 2012 and December 2022 at the Christian Medical College (CMC), Vellore, India. CMC Vellore is a 2,234-bed, multispecialty, tertiary-care teaching hospital that serves as a referral center for various medical disciplines. Data were retrieved from the hospital’s electronic medical records system (Clinical Workstation). Clinical information was collected for patients admitted with *Salmonella* Enteritidis bloodstream infections (BSI), including sociodemographic details, presenting clinical features, co-infections, antibiotic use, and patient outcomes. Additionally, underlying conditions such as HIV status, autoimmune disorders, chronic diseases, malignancies, the use of steroids, cytotoxic drugs, and other relevant comorbidities were documented. Patient outcomes including length of hospital stay, discharge status, and mortality rates were also assessed. All the patient identifying variables in the data were removed before the analysis.

### Bacterial isolates, identification and antimicrobial susceptibility testing (AST)

Archived *S*. Enteritidis bloodstream infection (BSI) isolates (n = 101), collected between 2012 and 2022, were revived by culturing. Eight representative isolates from other clinical specimens (Faeces, Pus etc) were included in the study for the purpose of comparison. Isolates originating from poultry specimen were included to investigate potential zoonotic transmission. From poultry samples (rectal swabs and droppings), *Salmonella* sp. was selectively enriched by culturing in buffered peptone water and individual colonies were isolated using MSRV (Modified semi-solid Rappaport-Vassiliadis) media [18]. The isolates were further confirmed as *S*. Enteritidis by standard biochemical and sero-agglutination tests based on the Kauffmann-White scheme [19]. The isolates were further confirmed as *Salmonella* Enteritidis through seven-gene multilocus sequence typing (MLST) and comparison with the EnteroBase database (https://enterobase.warwick.ac.uk) [20].

AST (disk diffusion) was performed against ampicillin (10 μg), chloramphenicol (30 μg), sulfamethoxazole-trimethoprim (1.25/23.75 μg), ciprofloxacin (5 μg), and ceftriaxone (30 μg). Clinical breakpoints followed Clinical and Laboratory Standards Institute (CLSI) guidelines, 2022 [21]*. Escherichia coli* ATCC 25922 served as the quality control strain.

### DNA extraction and whole genome sequencing

Among the 101 bloodstream isolates identified during the study period, 48 clinical *S*. Enteritidis isolates were successfully revived from the archived collection and selected for WGS. The remaining isolates could not be included because of non-revival, or other technical constraints. Additionally, 17 poultry-derived isolates were included, resulting in a final set of 65 isolates for WGS. Genomic DNA was extracted using a Wizard DNA purification kit (Promega, Madison, USA) as per the manufacturer’s instructions. Paired-end fragment libraries were prepared using a Nextera XT DNA sample preparation kit following the manufacturer’s instructions (Illumina, Inc., San Diego, USA). The pooled libraries were diluted at a final concentration of 1.8 pM and sequenced on an Illumina Nextseq 500 platform with 150-cycle paired-end chemistry.

### Genome assembly, annotation and genotyping

Sequencing reads were evaluated with FastQC v0.12.0 (https://www.bioinformatics.babraham.ac.uk/projects/fastqc/) and reads with low sequencing quality were trimmed and removed using Trimmomatic v0.39 (https://github.com/usadellab/Trimmomatic). High-quality reads were subjected to genome assembly using SKESA v.2.4.0 (https://github.com/ncbi/SKESA). The assemblies were analysed for contamination using Kraken v2.1.3 (https://github.com/DerrickWood/kraken2) and quality was assessed via QUAST v5.2.0 (https://github.com/ablab/quast). Draft genome assemblies were examined using Seqsero v2.0 (https://github.com/denglab/SeqSero2) to confirm the antigenic profile of the serotype [22]. Sequence types were assigned *in silico* for all the isolates using the Multilocus sequence typing (MLST) pipeline available in the Center for Genomic Epidemiology (CGE) (https://cge.food.dtu.dk/services/MLST/). Antimicrobial resistance genes were screened using NCBI AMRFinderPlus v3.11.20 (https://github.com/ncbi/amr). Plasmids were identified from the genome data by searching against the PlasmidFinder database (https://cge.food.dtu.dk/services/PlasmidFinder/). The presence of pSEN-like plasmids and their associated genes was identified by performing BLAST comparisons against reference plasmids pSEN (HG970000) and pSENV (JN885080). Genome annotations were performed using Prokka v1.14.5 (https://github.com/tseemann/prokka) based on a custom genus database (https://github.com/tseemann/prokka?tab=readme-ov-file#crazy-person).

### Pangenome derived phylogeny

Annotated genomes (GFF3 files) generated by Prokka comprising study isolates (n = 65) and a global representation of *S*. Enteritidis (n = 420) were used as input to evaluate pan-genome diversity using Panaroo v1.3.4 (https://github.com/gtonkinhill/panaroo) [23]. Panaroo was run with standard parameters with ‘remove invalid genes’ enabled. Subsequently, single nucleotide polymorphisms (SNPs) were extracted from the core gene alignment generated by Panaroo using SNP-sites (http://sanger-pathogens.github.io/snp-sites/). A Maximum likelihood (ML) tree was constructed using IQTree2 (TVM + F + ASC + R2; bootstrap replicates = 100) [24] and visualized with iTOL (https://itol.embl.de/). Subsequently, distantly related outgroup isolates belonging to diverse sequence types were removed and a phylogenetic tree was reconstructed (n = 475) to improve the cladistic structure. Phylogenetic clusters were assigned using rhierBAPS (https://github.com/gtonkinhill/rhierbaps) in its default mode (two cluster levels with 30 initial clusters). Minimum spanning trees (MSTs) were created and visualized using GrapeTree (https://achtman-lab.github.io/GrapeTree/MSTree_holder.html) from the core genome phylogeny [25]. Tree nodes were positioned through dynamic rendering and node style was adjusted by fine-tuning the node size and kurtosis. Nodes were coloured by the source of the isolates and node sizes were drawn proportionally to the number of isolates.

### Bayesian Inference analysis

Genome assemblies belonging to the Global epidemic and the Global Intermediate clades were aligned to the *S*. Enteritidis reference strain P125109 (GenBank: CP063700.1). Variant calling and alignment were performed using Snippy v4.6.0 (https://github.com/tseemann/snippy), and recombination was removed with Gubbins v3.4 (https://github.com/nickjcroucher/gubbins) using default parameters. The resulting recombination-filtered core alignment (5,390 SNPs) was used to infer time-scaled phylogenies.

To infer temporal evolutionary dynamics, Bayesian time-scaled phylogenies were generated using BEAST X v10.5 (https://github.com/beast-dev/beast-mcmc), following the analytical framework of Feasey et al. (2016) [13]. The GTR substitution model with gamma-distributed rate variation (four categories), a relaxed log-normal molecular clock, and a constant-size coalescent prior were applied. Default priors were retained. Two independent chains were run for 750 million MCMC generations, sampling every 1,000 steps, until all parameters achieved ESS > 200, as assessed in Tracer v1.7.2. A 10% burn-in was removed prior to summarizing the posterior distribution into a maximum clade credibility (MCC) tree using TreeAnnotator v10.5.0 (with median node heights). Temporal signal was evaluated by regressing root-to-tip genetic distances against sampling dates in TempEst v1.5.3. The dataset demonstrated a strong linear relationship (r² = 0.76), indicating a robust temporal structure suitable for molecular-clock dating. The residual mean (−0.000; 95% HPD: −0.001 to 0.001) and a Pearson correlation coefficient of 1.00 (95% HPD: 0.999–1.000) confirmed the absence of temporal bias and validated the suitability of the data for BEAST-based evolutionary inference.

### Pangenome clustering

The gene presence or absence matrix created by Panaroo was clustered according to the four major phylogroups using the twilight analysis package (https://github.com/ghoresh11/twilight) using the default thresholds [26]. The pan genome reference FASTA file (pan_genome_reference.fa) generated by Panaroo, which encompasses all the genes within the dataset, was clustered using CD-HIT-EST. [27] with a 90% identity and 90% sequence length coverage. Finally, clustered phage-associated genes were predicted using PHASTER database (https://phaster.ca/) using default settings [28]. The prophage regions were annotated using Bakta v1.11.4 (https://github.com/oschwengers/bakta) [29] and classified by functional categories using COGclassifier (https://github.com/moshi4/COGclassifier). The predicted prophage regions were then manually curated to determine their gene composition and structural organization. Interactive visualization of the phylogenetic tree and PHASTER summary data was carried out in Phandango v1.3.0 [30].

### Lineage wise mutation profiling

Mutations were identified using *in-silico* analysis of single nucleotide polymorphisms (SNPs) using Snippy v4.6.0 mapping and variant calling pipeline (https://github.com/tseemann/snippy). To obtain the SNPs, we mapped the draft genomes (n = 475) against the annotated features of the reference genome P125109 (NC_011294.1). We utilized custom-written bash scripts to extract the pattern of mutation accumulation with respect to the phylogenetic lineages. Genes that contained either frameshift mutation or a premature stop codon were manually curated and classified as hypothetically disrupted coding sequences (HDCS) or pseudogenes.

### Invasiveness index calculation

The potential of *S*. Enteritidis to cause invasive disease was calculated based on a machine learning pre trained invasive index predictive model (https://github.com/Gardner-BinfLab/invasive_salmonella) [31]. This approach classifies the genomes of invasive and gastrointestinal *Salmonella* by scoring deleterious mutations in 196 top predictor genes. The distribution of invasiveness index values for four major phylogroups was compared using the Kruskal-Wallis H Test.

### Statistical analysis

Statistical analysis was performed to investigate the potential association between severe immunosuppression and the clinical characteristics of *S*. Enteritidis infection. Data analysis was performed using GraphPad Prism 5.0 for Windows (GraphPad Software Inc). Continuous variables were represented as median (IQR). Categorical variables were presented as either numbers or percentages. To compare these categorical variables, we employed either Fisher’s exact test or the Chi-squared test, as appropriate for the specific analysis. We calculated the odds ratio (OR) along with its corresponding 95% confidence intervals (CI). Statistical significance was determined by a P-value < 0.05 (two-tailed).

## Results

### Infants and immunosuppressed are more susceptible to *S*. Enteritidis BSI

The patient records indicated a total of 506 patients diagnosed with blood culture-positive iNTS disease between January 2012 and December 2022. Of these, 131 cases were confirmed to be caused by *S*. Enteritidis by conventional microbiological methods. After excluding 30 cases due to the absence of relevant clinical data and duplicate isolates (repeated samples from the same patient within 14 days), hospital records for 101 *S*. Enteritidis bacteraemia cases were analysed. The median age of affected patients was 34.5 years (IQR: 9–54.5 years) (Table 1). Pediatric patients (<16 years) accounted for 27.72% (28/101) of cases, with infants (<12 months) comprising 13.86% (14/101), indicating that a substantial proportion of BSI occurred in younger age groups. Compared with immunosuppressed patients, pediatric cases were more common among non-immunosuppressed patients (44.18% vs. 15.52%; p = 0.003; OR = 0.23, 95% CI: 0.09–0.59). The most common presenting symptoms on admission included fever (75.25%), anemia/unhealthy appearance (59.41%), diarrhea (35.64%), and cough (30.69%). Among 86 inpatients, the median fever duration was 6 days (IQR: 3–10 days). Male patients accounted for 65.35% (66/101) of cases.

**Table 1:** Comparison between NTS bacteraemia patients with and without Immunosuppressive disease conditions.

Variable	Overall n (%)/median (IQR) (n = 101)	Immunosuppressive conditions n (%)/median (IQR)		p-value	OR	95% CI
		Yes (n = 58)	No (n = 43)			
Sex (Male)	66 (65.34)	36 (62.1)	30 (69.76)			
Age	34.5 (9 – 54.5)	36 (25 – 50)	24.5 (0 – 55.75)			
Pediatric (<16 years)	28 (27.72)	9 (15.52)	19 (44.19)	0.003	0.23	0.09-0.59
Duration of fever	6 (3 – 10)	7 (4 - 11)	5 (1 – 8)			
Co-infection	18 (17.82)	11 (18.96)	7 (16.28)	0.797	1.2	0.42–3.41
Antibiotic combination therapy	66 (65.34)	38 (65.52)	28 (65.12)	1	1.02	0.44–2.33
Abdominal pain	27 (26.73)	16 (27.58)	11 (25.58)	1	1.11	0.45–2.71
Ascites	12 (11.88)	9 (15.52)	2 (4.65)	0.111	3.77	0.77–18.42
Cough	31 (30.69)	18 (31.03)	13 (30.23)	1	1.04	0.44–2.44
Diarrhea	36 (35.64)	19 (32.76)	17 (39.53)	0.532	0.75	0.33–1.69
Fever	76 (75.25)	46 (79.31)	30 (69.77)	0.352	1.66	0.67–4.12
Pneumonia	22 (21.78)	14 (24.14)	8 (18.6)	0.628	1.39	0.52–3.69
Septic Shock	20 (19.8)	16 (27.59)	4 (9.3)	0.025	3.71	1.14–12.08
Unhealthy/ Anaemia	60 (59.41)	38 (65.52)	22 (51.16)	0.158	1.81	0.81–4.06
Death	11 (10.89)	8 (13.79)	3 (6.98)	0.346	2.13	0.53–8.57

We examined the impact of immunosuppressive conditions on *S*. Enteritidis bacteremia by comparing patients with these conditions to those without them. Among the 101 patients with *S*. Enteritidis bacteremia, 57.42% (58/101) had immunosuppressive conditions, including cancer/malignancy (24.75%), autoimmune disorders (21.78%), HIV infection (8.91%), and chronic steroid or chemotherapy use (39.6%) (Table 2). Among non-immunosuppressed patients (n = 43), the pediatric population was disproportionately affected (44.18%), further emphasizing the vulnerability of children. Septic shock was significantly more frequent in immunosuppressed patients (27.59% vs. 9.3%; p = 0.025; OR = 3.71, 95% CI: 1.14-12.08), indicating a higher risk of severe disease outcomes.

**Table 2:** Clinical characteristics of patients with *S*. Enteritidis bacteraemia.

	Characteristics	No. of cases (n = 101)	%
**Underlying conditions**			
Immunosuppressive condition (n = 58)	HIV infection	9	8.91
	Hepatitis infection	5	4.9
	Autoimmune disorders	22	21.78
	Cancer/Malignancy	25	24.75
	Immunosuppressives (Steroid therapy/ Chemotherapy)	40	39.6
Other conditions (n = 43)	Heart disease	10	9.9
	Hypertension	23	22.77
	Diabetes	19	18.81
	Renal disease	12	11.88
	Surgery/invasive procedure	14	13.86

To evaluate treatment strategies and patient outcomes, we analyzed antibiotic therapy and recovery rates among affected individuals. A total of 65.34% (66/101) of patients received more than one antibiotic, primarily consisting of β-lactams (carbapenems/ third-generation cephalosporins/ β-lactam/β-lactamase inhibitors) and azithromycin. Clinical outcomes showed an overall recovery rate of 83.17% (84/101), while 10.89% (11/101) of patients died. Although mortality was higher in immunosuppressed patients (13.79%) compared to non-immunosuppressed patients (6.98%), this difference was not statistically significant (p = 0.346; OR = 2.13, 95% CI: 0.53-8.57).

### Phenotypic characterization

*S*. Enteritidis (n = 65) strains were revived from the archived collections at the Department of Clinical Microbiology, CMC Vellore. Study isolates that were confirmed to be *S*. Enteritidis by biochemical testing and serotyping were subjected to AST. The results demonstrated that all isolates were susceptible to first-line antibiotics, (ampicillin, chloramphenicol, trimethoprim/sulfamethoxazole), and ceftriaxone. However, there was a decrease in susceptibility to fluoroquinolones, with 26.1% (17/65) of the isolates exhibiting non-susceptibility to ciprofloxacin (S1 Table).

### Population structure of *S*. Enteritidis consists of four major phylogenetic clades

The phylogenetic tree of 486 genomes was established by sequencing 65 isolates of *S*. Enteritidis (both clinical and poultry) and 421 publicly available genome sequences (S1 Fig). The metadata of isolates selected were described in S2 Table. To improve branch length resolution, outgroup genomes belonging to diverse sequence types (ST180, ST1975, ST3304) were excluded, resulting in an abbreviated ML tree of 475 genomes. This dataset included 59 study isolates, 42 from clinical sources and 17 from poultry along with genomes retrieved from public databases, allowing for a detailed assessment of the population structure of *S*. Enteritidis.

**Fig 1:**
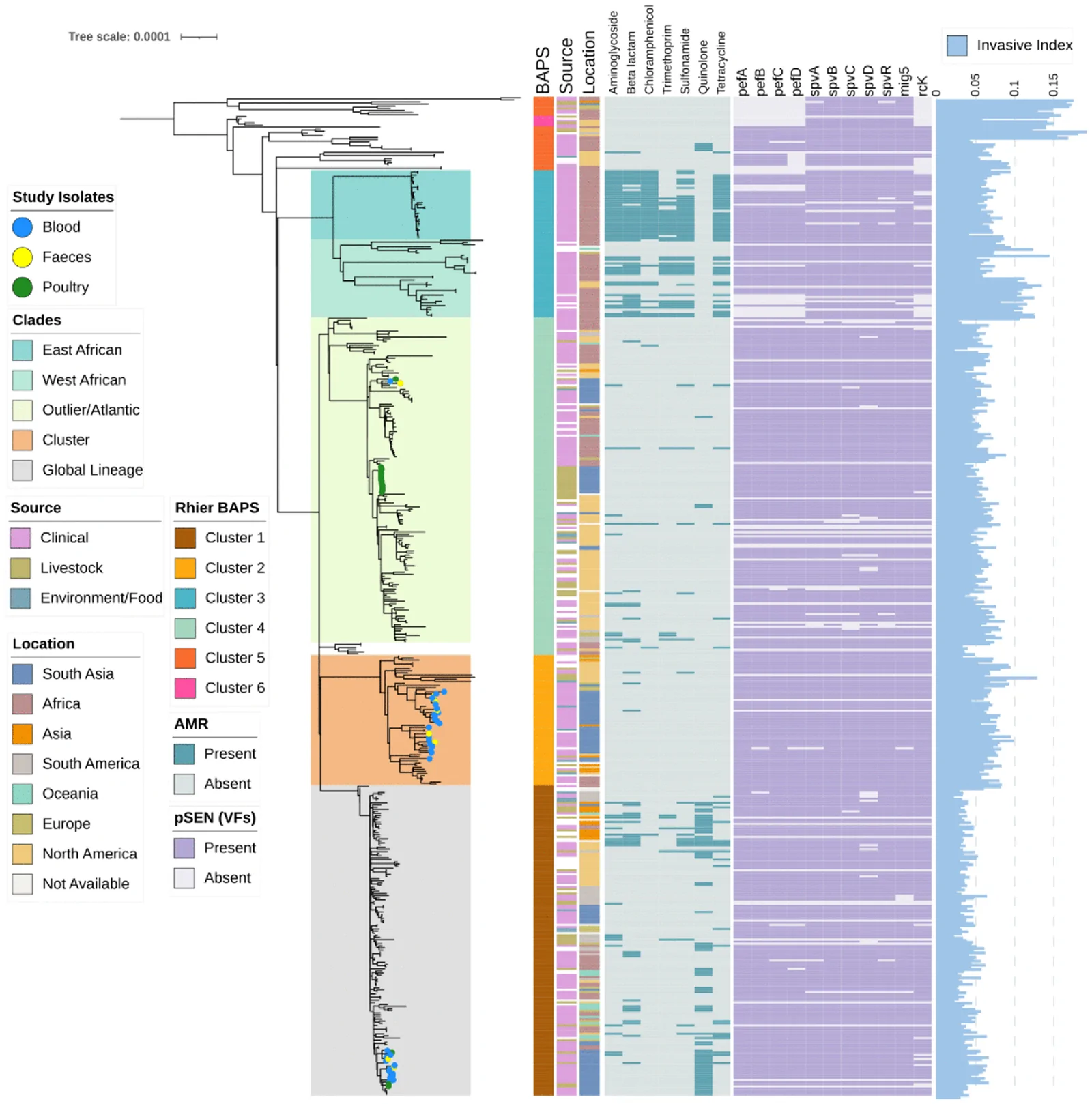
Maximum likelihood phylogenetic tree of *Salmonella* Enteritidis was constructed using 13,876 SNPs from 3,661 core genes (>95% presence) identified through pan-genome analysis with Panaroo (https://github.com/gtonkinhill/panaroo). The midpoint-rooted phylogeny was inferred using IQ-TREE2 (http://www.iqtree.org/), with bootstrap support values calculated from 100 replicates. The analysis included 475 *S*. Enteritidis genomes, of which 59 were isolates from this study. The phylogenetic tree reveals the distribution of *S*. Enteritidis within four main clusters, corresponding to distinct phylogroups. Study isolates are highlighted with red branch symbols. Metadata annotations are displayed as color strips: (1) BAPS clusters, (2) source of isolates, and (3) geographic location. Resistance profiles and key virulence genes are represented as heatmaps, while the invasiveness index for each genome is plotted as an external bar plot. The scale bar indicates substitutions per site. The tree was visualized and annotated using iTOL (https://itol.embl.de/).

Phylogenetic analysis based on 13,876 core gene SNPs revealed that *S*. Enteritidis genomes clustered into four major phylogenetic clades (Fig 1). Clustering using RhierBAPS (Level 1) identified six BAPS clusters, four of which corresponded to the major phylogroups. In addition to previously defined groups namely the African lineage (Cluster 3), the Global outlier/Atlantic lineage (Cluster 4), and the Global epidemic clade (Cluster 1), we identified a distinct phylogroup (Cluster 2), separated by 163 unique SNPs (S3 Table). This novel cluster, which displays a geographically widespread global distribution, is hereafter referred to as the ‘Global Intermediate Clade’. Phylogenetically, the Global Intermediate Clade forms a distinct monophyletic lineage positioned between the Global outlier/Atlantic and Global Epidemic clades, indicating a shared ancestral origin with both.

The study isolates were distributed between the Global Intermediate Clade (44.07%; 26/59), Global epidemic clade (28.8%; 17/59) and Global outlier clade (27.12%; 16/59). Notably, the majority of isolates were assigned to Sequence Type (ST)11, irrespective of their phylogenetic classification. Additionally, single-locus variants (SLVs) of ST11, including ST745, ST1479, and ST1974, were identified across the four primary clades. The geographic distribution of *S*. Enteritidis phylogroups revealed distinct regional patterns. While some lineages exhibit global distribution, others appear geographically restricted. For instance, the African lineage includes West African and East African sub-lineages, both associated with iNTS disease. In contrast, the Global outlier and Global epidemic clusters contain isolates from diverse sources and geographic regions, spanning collections from 1925 to 2024 (S4 Table).

To further elucidate the evolutionary history of the major phylogroups, we reconstructed a Bayesian time-calibrated phylogeny focusing on the Global Intermediate and Global Epidemic clades (S2 Fig). The BI analysis estimated that the most recent common ancestor (MRCA) of the Global Epidemic Clade emerged around 1762 AD (95% HPD: 1607–1921), consistent with a lineage that has undergone substantial expansion during the modern era. In comparison, the Global Intermediate Clade was inferred to have originated slightly later, around 1789 AD (95% HPD: 1692–1941). This lineage displayed considerable genetic heterogeneity, with multiple deeply branching sublineages, indicating long-term evolutionary stability and slower diversification compared with the rapidly expanding Global Epidemic Clade. The earliest sequenced representative dates to 1950, with additional isolates from Europe and Asia confirming its global circulation for several decades.

### *S*. Enteritidis isolates from poultry were genetically distinct from those causing BSI

Comparative genome analysis was performed to investigate the genetic relationship between human and livestock isolates of *S*. Enteritidis and to assess potential zoonotic transmission pathways. Among the poultry isolates from this study, fourteen (n = 14/17) grouped within the Global outlier cluster, forming a distinct subcluster that was genetically separate from most clinical isolates (S3 Fig). Three remaining poultry isolates clustered within the Global Epidemic clade, while none were associated with the Global Intermediate clade. However, the presence of livestock isolates from global datasets within the clinical clusters, particularly Cluster 2, suggests that animal reservoirs may still contribute to the broader transmission dynamics of *S*. Enteritidis, although direct evidence from this study remains limited. (Fig 1).

### Limited antimicrobial resistance and plasmids among Indian *S*. Enteritidis

Among the *S*. Enteritidis study isolates, we identified only a few genetic determinants of acquired antibiotic resistance. With the exception of one isolate, none of the study isolates contained detectable AMR genes, indicating a low prevalence of acquired resistance within this dataset. To provide a broader context, phylogroup-wise AMR screening was conducted on a global collection of isolates. Multidrug resistance (MDR) markers, including *bla*_TEM_, *cat*, *sul*, and *dfrA*, which confer resistance to first-line antibiotics, were primarily associated with East and West African clades. In contrast, AMR determinants showed variable and generally lower prevalence in other phylogenetic groups, regardless of their geographic origin. Apart from occasional ampicillin and co-trimoxazole resistance markers detected within the Global Epidemic Lineage, AMR genes were largely absent in all non-African clades. With respect to chromosomal-mediated quinolone resistance, most *S*. Enteritidis isolates remained susceptible to fluoroquinolones. However, *gyrA* mutations (S83Y or D87G) associated with quinolone resistance were detected in 23.1% (15/65) of isolates, all of which belonged to the Global Epidemic Clade, indicating the emergence of fluoroquinolone resistance within this lineage (Fig 1).

The pSEN-like virulence plasmid (pSENV) was commonly detected among global *S*. Enteritidis isolates. As expected, all isolates belonging to the East African sub-clade carried the pSENV plasmid and its associated replicon. In other phylogenetic clades, a small number of isolates lacked the plasmid, although this appeared sporadic and not associated with lineage structure (S4 Fig). Further analysis showed that core pSENV-mediated virulence factors typical of *S*. Enteritidis including fimbrial adhesion genes (*pefABCD*), T3SS effector genes (*spvBCD*), and additional virulence genes (*mig-5* and *rck*) were widely present across isolates (Fig 1). Notably, the newly defined Global Intermediate Clade showed a particularly high prevalence of these plasmid-associated determinants, with 93.5% (58/62) of isolates carrying all 11 genes, highlighting the potential contribution of this lineage to plasmid-mediated pathogenicity.

### African and intermediate clade contained high levels of genome degradation

Frameshift mutations and premature stop codons leading to hypothetically disrupted coding sequences (HDCS) were analyzed across major phylogenetic clusters in the dataset (S5 Table). Genomes within the African cluster, including both West African and East African sub-lineages, exhibited the highest number of HDCS (n = 9) compared to isolates from the Global Epidemic and Global outlier clusters (n = 2). Additionally, representative genomes from the East African cluster contained an extra 16 HDCS, although none were associated with virulence genes. Isolates belonging to the Global intermediate clade were distinguished by eight lineage-defining HDCS (S5 Table).

### Global Intermediate clade has the second-highest invasiveness index, following the West African clade

**Fig 2:**
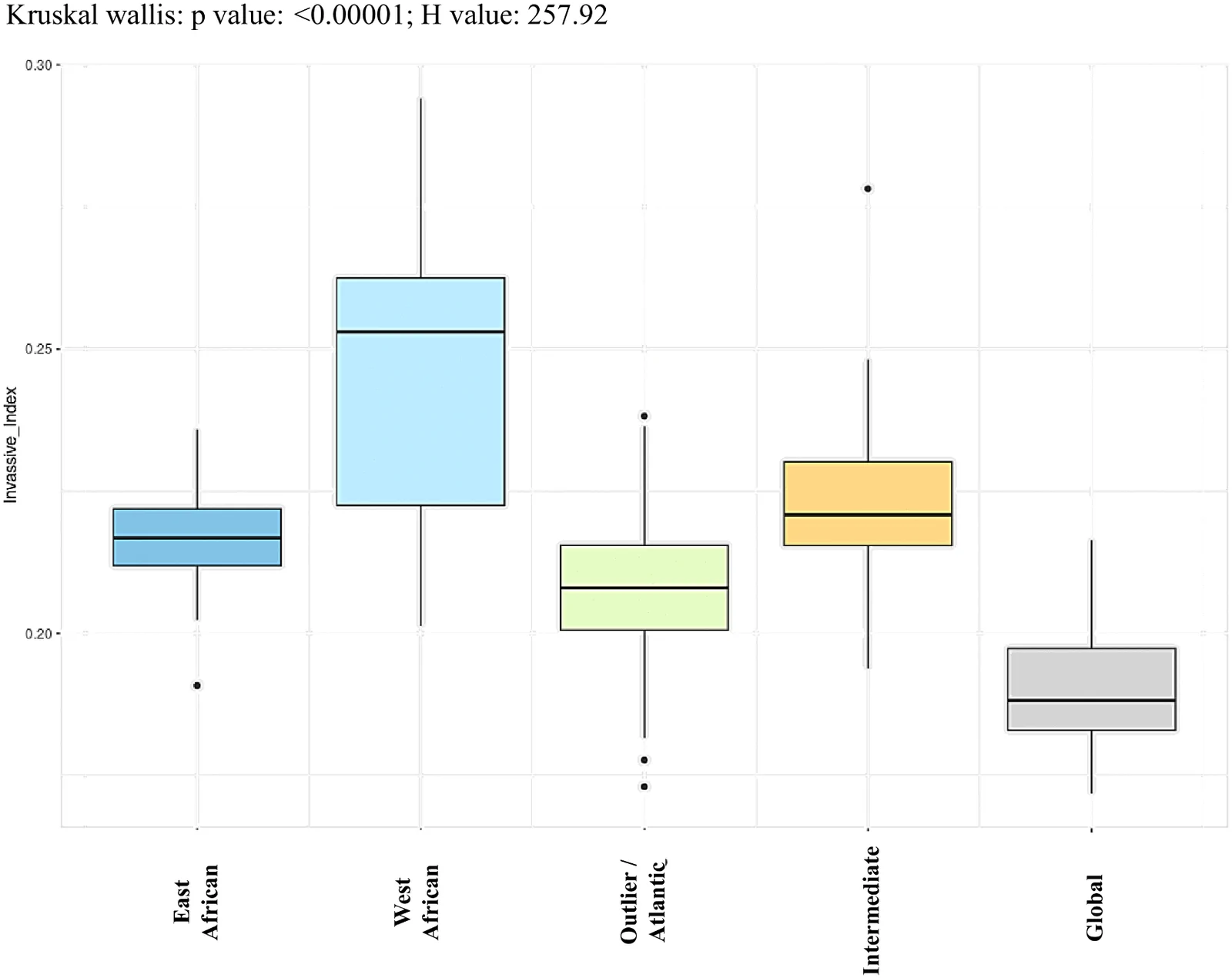
The lineage wise invasiveness index for all *Salmonella* Enteritidis genomes analyzed in this study was determined using the approach described by Wheeler et al. The median and standard deviation (SD) values for each lineage are as follows: East African (median = 0.217, SD = 0.010), West African (median = 0.253, SD = 0.026), Global outlier (median = 0.208, SD = 0.011), Global Intermediate (median = 0.221, SD = 0.013), and Global Epidemic (median = 0.188, SD = 0.010). Mann-Whitney U-test was used to compare differences between groups. In the boxplot, boxes represent the interquartile range (IQR), whiskers extend to 1.5 × IQR, and outliers are shown as points. The Kruskal-Wallis test revealed significant differences across lineages (p < 0.0001, H = 257.92).

The invasiveness of *S.* Enteritidis phylogroups was evaluated using machine-learning based Invasive index calculator. This pipeline utilizes a random forest classifier model to produce predictive scores, denoted as delta bitscores (DBS). It accomplishes this by examining mutations within a previously defined set of 196 genes, as explained earlier. The potential of different phylogroups to cause extra-intestinal infection was compared using Wilcoxon–Mann–Whitney test. The results indicate that all examined *S.* Enteritidis phylogroups remain to be gastrointestinal (DBS < 0.5). Nonetheless, the invasiveness index varies among these phylogroups (Fig 2). We observed a high invasiveness index (median  =  0.253, SD  =  0.026) for isolates belonging to West African clade while the global epidemic clade had the lowest (median  =  0.188, SD  =  0.01). The global intermediate clade also exhibited a relatively high invasiveness index (median 0.221, SD = 0.013) compared to East African and outlier clusters.

### Pan-genome clustering suggests the evolution of *S*. Enteritidis is largely driven by the acquisition of prophage genes

Pan-genome analysis of *Salmonella* Enteritidis, excluding highly divergent phylogroups (e.g., ST1975), identified 8,098 gene families. The core genome, comprising genes present in >99% of analyzed genomes, consisted of 3,672 genes, representing 73.8% of the total 5,344 genes. Analysis of the accessory genome revealed distinct gene family distribution patterns among established phylogroups (S5 Fig).

**Fig 3:**
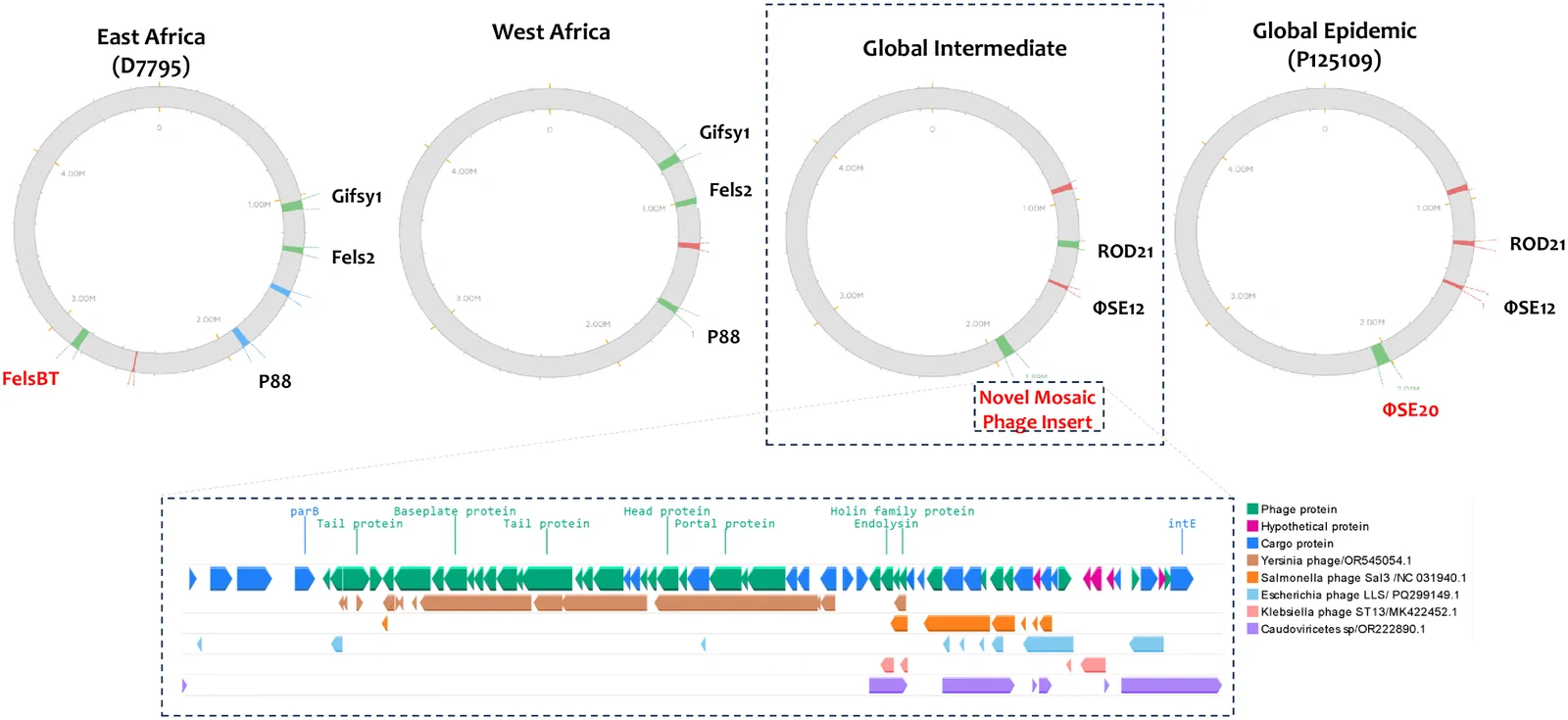
Prophage Map of Major *Salmonella* Enteritidis Lineages. The position of prophage regions identified in representative *S.* Enteritidis ST11 strains from each major lineage using PHASTER (https://phaster.ca/). The panel illustrates the genetic organization of a mosaic prophage identified in the global intermediate clade. Comparative genomic analysis of this mosaic prophage was performed using Proksee (https://proksee.ca/), showing its similarity to closest prophage homologs *Yersinia* phage vB_YpM (OR545054.1), *Salmonella* phage 118970 Sal3 (NC_031940.1), *Escherichia* phage LLS (PQ299149.1), and *Klebsiella* phage ST13-OXA48phi12 (MK422452.1) and Caudoviricetes sp. isolate MSP0459 (OR222890.1).

Lineage-specific gene gain and loss were most evident in the Global Intermediate Clade, which harbors a unique 48.2-kb mosaic prophage that is absent from all other *S*. Enteritidis phylogroups (Fig 3). Comparative genome analysis revealed that this prophage is composed of two major genomic modules, each exhibiting homology to prophage remnants previously described in diverse Enterobacterales. This architecture reflects a chimeric origin formed through modular acquisition. The larger module displayed the highest similarity to *Yersinia* phage vB_YpM (OR545054.1), while the second module showed closest correspondence to Caudoviricetes sp. isolate MSP0459 (OR222890.1). Additional partial matches to *Salmonella* phage 118970 Sal3 (NC_031940.1), *Escherichia* phage LLS (PQ299149.1), and *Klebsiella* phage ST13-OXA48phi12 (MK422452.1) were also detected within segments of module 2. The modular alignment pattern demonstrates that these phage-derived regions are co-localized within a single genomic island, a configuration characteristic of sequential recombination and mosaic assembly through phage modular exchange.

Among the 59 isolates assigned to the Global Intermediate Clade, 45 genomes (76.3%) harbored both modules, whereas a subclade of 14 genomes (23.7%) retained only the first module. In these isolates, the Caudoviricetes-associated region was completely absent, suggesting lineage-specific reductive evolution or progressive modular loss within the clade (S6 Fig). Annotation of this prophage region identified 66 coding sequences (CDS), of which 32 CDS were assigned to COG functional categories (S6 Table). As expected for a phage-derived genomic island, the majority of functionally assigned CDS belonged to COG category X (Mobilome: prophages, transposons). Additional functions included replication and recombination proteins, cell envelope-associated genes, and metabolic or stress-associated components. The co-occurrence of phage modules and host-derived segments indicates that this prophage represents a highly modular, recombination-rich genomic island, potentially acquired through successive horizontal exchange events. This mosaic prophage is a defining genomic feature of the Global Intermediate Clade and may contribute to the lineage’s distinct ecological behaviour and its strong association with extra-intestinal infections.

## Discussion

iNTS infections are increasingly recognized as an important public health concern in low- and middle-income countries (LMIC), yet their epidemiology outside sub-Saharan Africa remains comparatively underexplored [3,6,32,33]. In India, *S*. Enteritidis is the second most common iNTS serovar causing BSI, with previous studies also documenting its involvement in foodborne outbreaks [17,34,35]. This study provides the first comprehensive genomic and clinical assessment of invasive *S*. Enteritidis in India, thereby expanding the global understanding of iNTS population structure and disease dynamics.

While iNTS disease in Africa is strongly associated with HIV infection, malnutrition, and malaria co-infection [6,14,36,37], our findings reveal a distinct epidemiological pattern in India. Here, invasive *S*. Enteritidis infections were primarily linked to non-HIV forms of immunosuppression, including malignancy, autoimmune disorders, and chronic steroid or chemotherapy use. A substantial proportion of cases also occurred in infants, consistent with the recognized susceptibility of individuals with immature or compromised immune systems. These observations align with broader evidence indicating that host immune status rather than specific comorbidities plays a central role in predisposing individuals to iNTS disease [33,38]. Thus, the Indian data highlight how regional epidemiological contexts shape iNTS risk profiles, and suggest that factors prominent in African settings may not fully explain *S*. Enteritidis invasiveness in all geographic regions.

Globally, *S*. Enteritidis displays striking genetic homogeneity dominated by sequence type ST11, yet several phylogenetically distinct clades have emerged through adaptation to different ecological niches and host environments [13,39,40]. Within this broader landscape, our study identifies a previously unrecognized lineage ‘the Global Intermediate Clade’ whose most recent common ancestor (MRCA) is estimated to have originated around 1789 AD. The MRCA dating suggests that this lineage is not a recent evolutionary emergence but rather a long-standing component of the global *S*. Enteritidis population that has only recently become epidemiologically prominent in India. Comparable patterns have been observed in the African invasive lineages, whose origins trace back to the early–mid 20th century (e.g., East African clade: 1945 (95% HPD: 1924–1951) and West African clade: 1933 (95% HPD: 1901–1956) [13]. These parallels suggest that long-term cryptic circulation followed by regional expansion may be a recurring evolutionary trajectory for major *S*. Enteritidis lineages.

From an evolutionary perspective, the identification of the ‘Global Intermediate Clade’ reinforces the notion that *S*. Enteritidis comprises a multi-lineage population structure in which lineages differ in ecological associations and pathogenic potential. Although pinpointing the precise evolutionary steps that drive a shift toward extra-intestinal disease remains challenging, previous work in *S*. Typhimurium ST313 and *S*. Pullorum has shown that genome degradation, particularly in genes involved in virulence, metabolism and nutrient uptake, can facilitate reduced reliance on intestinal colonization and enhanced systemic survival [12,41]. Similar features characterize the African *S*. Enteritidis clades, which show pronounced genomic degradation and expanded virulence plasmids adapted for persistence in immunocompromised hosts [13]. The Global Intermediate Clade displays parallel signatures, including inactivation of metabolic and membrane transport genes and an invasiveness index comparable to African sublineages, suggesting functional shifts toward persistence in host cells. These patterns align with well-described mechanisms of host adaptation in other *Salmonella* serovars, where functional gene loss, pseudogene formation, and horizontal gene transfer frequently accompany niche specialization [41–44].

Prophage acquisition is a well-established driver of virulence diversification and host adaptation in *Salmonella* [45]. Previous studies in *S*. Enteritidis, *S*. Typhimurium, and *S*. Infantis have demonstrated how lineage-specific prophages shape ecological specialization and pathogenic potential [13,46–48]. In the Global Intermediate clade, we identified a distinctive mosaic prophage that contains genetic modules homologous to phages of *Salmonella, Yersinia, Escherichia, Klebsiella*, and Caudoviricetes, and several others. The organization of this prophage consistent with classical modular exchange, a mechanism through which temperate phages diversify and assemble new genomic architectures [45,49]. Our findings suggest that the prophage in the Global Intermediate clade likely originated through such modular exchange events. Given that this prophage is restricted to the Global Intermediate Clade and present in near-complete form in most isolates, its presence likely contributed to the emergence, ecological differentiation, and lineage-specific adaptation of this clade [50].

The relationship between invasive *S*. Enteritidis and poultry-associated strains remains complex. Historically, global dissemination of *S*. Enteritidis has been driven by industrial poultry production and egg-associated transmission, leading to the dominance of the global epidemic clade in foodborne outbreaks [16,51]. In our study, Indian poultry isolates clustered within the Outlier and Epidemic clades and were genetically distinct from human bloodstream isolates belonging to the Global Intermediate clade. This decoupling suggests that the invasive phenotype present in India is not currently maintained in the local poultry reservoir. Nevertheless, livestock-associated genomes from global datasets appear in related phylogenetic clusters, indicating that historical transmission from animal reservoirs cannot be ruled out. These findings underscore the importance of expanded One-Health genomic surveillance to clarify transmission pathways and assess the risk of reintroduction of invasive lineages into foodborne reservoirs [52].

These observations have important implications for public health and disease control. The identification of an invasive lineage not currently linked to the poultry reservoir suggests that surveillance systems centered solely on foodborne transmission may fail to detect clinically significant lineages. Routine incorporation of WGS into national enteric disease programs integrated across human, veterinary, and environmental sectors would enable earlier detection of emerging invasive lineages, more accurate risk assessment, and targeted interventions [53]. These data also emphasize the need for region-specific strategies in global *S*. Enteritidis control efforts, particularly in settings where the drivers of invasiveness may differ from those historically described.

Our study has several limitations. First, clinical data originate from a single Indian hospital, limiting generalizability to national trends. Second, many clinical variables lacked statistical association with outcomes. Third, the phylogenetic analysis included 65 of 101 isolates from the study period, and poultry isolates were underrepresented, restricting ecological inference. Finally, functional characterization of the mosaic prophage, though beyond the scope of this study, is needed to elucidate its biological and evolutionary significance. Despite these limitations, our findings provide a robust phylogenomic framework for understanding an understudied pathogen and highlight the emergence of a distinct invasive lineage of *S*. Enteritidis in South Asia.

## Conclusion

This study provides the first integrated clinical and genomic analysis of *S*. Enteritidis from India, showing that infections predominantly affect individuals with impaired immunity, consistent with the opportunistic nature of iNTS disease. Phylogenomic reconstruction identified a previously unrecognized lineage ‘the Global Intermediate Clade’ characterized by distinct prophage content and signs of genomic degradation. Although this clade showed a comparatively elevated invasiveness index, this metric reflects genomic signatures rather than a direct measure of clinical virulence and should be interpreted cautiously. The genetic separation between invasive isolates and contemporary poultry strains underscores the need for expanded One-Health surveillance to clarify reservoirs and transmission pathways. Further work including functional analyses, expanded animal/environmental sampling, and longitudinal genomic surveillance will be essential to clarify the evolutionary drivers and epidemiological significance of this emerging lineage.

## Supporting information

S1 Fig Pan-genome based core genome phylogenetic tree of 486 *S*. Enteritidis showing the comparative phylogenetic clustering by MLST. Study isolates are highlighted with red branch symbols. MLST are displayed as color strips. The tree was visualized and labeled using iTOL (https://itol.embl.de/).
(TIFF)

S2 Fig (a) Bayesian time-scaled phylogeny of the Global Epidemic and Global Intermediate clades of *Salmonella* Enteritidis. A Bayesian maximum clade credibility (MCC) tree reconstructed from 5,390 recombination-filtered core SNPs, showing the temporal evolution of genomes belonging to the Global Epidemic and Global Intermediate clades of *S*. Enteritidis. The analysis was performed in BEAST X v10.5 under a GTR+GAMMA substitution model, a relaxed log-normal molecular clock, and a constant-size coalescent prior. Node bars indicate the 95% Highest Posterior Density (HPD) intervals for divergence time estimates. Branch lengths are scaled in calendar years. Colours denote major phylogenetic groups as indicated. (b) Histogram showing the distribution of collection dates of all S. Enteritidis isolates. (c) Root-to-tip regression showing evidence of positive temporal signal.
(TIFF)

S3 Fig Minimum spanning tree (MST) of *S.* Enteritidis isolates (n = 475) constructed and visualized using GrapeTree (https://achtman-lab.github.io/GrapeTree/MSTree_holder.html). Each node represents a unique ST or Clonal group, and node size is proportional to the number of isolates with that ST. Node color indicates the source from which the isolate was obtained. Tree nodes were positioned through dynamic rendering and node style was adjusted by fine-tuning the node size and kurtosis.
(TIFF)

S4 Fig Phandango heatmap illustration (https://jameshadfield.github.io/phandango/#/) focusing on the presence of AMR genes, virulence genes and pSEN-like plasmid associated genes across phylogenetic lineages. Orange blocks indicates the presence of a gene while purple indicates its absence.
(TIFF)

S5 Fig Pangenome analysis of 475 *S.* Enteritidis isolates indicates the diversity in accessory genomes among the different phylogenetic lineages. Phandango heatmap illustration (Hadfield et al. 2018) of gene presence and absence matrix (right side) against Maximum likelihood phylogenetic tree (Left side) with clades correlate with the observation derived from phylogenetic analysis. Orange blocks indicates the presence of a gene while purple indicates its absence. A highlighted region (white dashed rectangle) indicates a cluster of lineage-specific genes.
(TIFF)

S6 Fig Phandango heatmap illustration (https://jameshadfield.github.io/phandango/#/) focusing on the presence of novel mosaic prophage region among isolates belonging to Global Intermediate Clade. Orange blocks represent the presence of the prophage modules, while purple indicates absence.
(TIFF)

S1 Table List of *S.* Enteritidis isolates included in the study, along with sequencing quality control metrics, genome assembly statistics, and antimicrobial susceptibility profiles. (XLSX)

S2 Table List of *S.* Enteritidis genomes incorporated into the global phylogeny, including isolate source, geographic origin (region and country), year of collection, MLST type, invasive index, RhierBAPS cluster assignments, and final lineage designation. (XLSX)

S3 Table List of 163 clade-specific SNPs distinguishing the Global Intermediate Clade from all other phylogenetic groups. (XLSX)

S4 Table List of *S.* Enteritidis genomes available in EnteroBase database that were assigned to the Global Intermediate Clade, including metadata. (XLSX)

S5 Table Summary of frameshift mutations and premature stop codons leading to highly degraded or hypothetically disrupted coding sequences (HDCS) conserved across the major *S.* Enteritidis global phylogenetic lineages. (XLSX)

S6 Table List of COG functional categories assigned to genes within the novel mosaic prophage of the Global Intermediate Clade. (XLSX)
